# The Association Between Loneliness and Cognitive Impairment among Older Men and Women in China: A Nationwide Longitudinal Study

**DOI:** 10.3390/ijerph16162877

**Published:** 2019-08-12

**Authors:** Zi Zhou, Fanzhen Mao, Wei Zhang, Samuel D. Towne, Ping Wang, Ya Fang

**Affiliations:** 1 State Key Laboratory of Molecular Vaccine and Molecular Diagnostics, School of Public Health, Xiamen University, Xiang’an South Road, Xiang’an District, Xiamen 361102, China; 2 Key Laboratory of Health Technology Assessment of Fujian Province University, School of Public Health, Xiamen University, Xiang’an South Road, Xiang’an District, Xiamen 361102, China; 3 Department of Health Management and Informatics, University of Central Florida, Orlando, FL 32816, USA; 4 Disability, Aging, and Technology Cluster, University of Central Florida, Orlando, FL 32816, USA; 5 Department of Environmental and Occupational Health, School of Public Health, Texas A&M University, College Station, TX 77843, USA

**Keywords:** loneliness, cognitive impairment, older adults, sex differences, China

## Abstract

We aimed to investigate the association between loneliness and cognitive impairment among older men and women in China. Data for 6898 eligible participants aged 65 years and older were derived from the latest two waves (2008/2009 and 2011/2012) of the Chinese Longitudinal Healthy Longevity Survey. A logistic regression analysis was performed to determine whether the association between loneliness at baseline and the risk of cognitive impairment at follow-up varied by sex, with adjustment for social-demographic variables, social isolation, lifestyles, and health status. The rates of baseline loneliness and follow-up cognitive impairment were both higher among women than men. Loneliness at baseline was significantly associated with cognitive impairment at follow-up among elderly men (OR = 1.30; 95% CI 1.01–1.69), even after adjusting for potential confounding variables; however, a similar association was not observed among elderly women (OR = 0.98; 95% CI 0.81–1.19). Multiple imputations were applied to address missing data. Although elderly women more frequently reported feelings of loneliness, the impact of loneliness on cognitive impairment was significant among elderly men but not elderly women. Interventions designed to decrease the incidence of loneliness may be particularly beneficial for the reduction of cognitive impairment among elderly Chinese men.

## 1. Introduction

The presence of cognitive impairment, which can be assessed based on one’s cognitive performance, may be associated with a rate of cognitive decline that is more rapid than the rate of decline seen among unimpaired persons and as such presents a strong possibility of progression to dementia [1]. Work that seeks to identify factors associated with the rate of cognitive decline is critical and timely, especially as Alzheimer’s disease and related dementia is estimated to affect approximately 152 million people by 2050, up from nearly 50 million in 2018 [2]. Further, the expected growth (in millions of persons with dementia) is projected to be substantially higher in low-to-middle income countries, as compared to high-income countries along the same timeline [3]. Thus, this is truly a global issue that is expected to increasingly affect those in settings with more limited resources (e.g., lower income countries). At present, there is no effective treatment available for cognitive impairment. Thus, identifying modifiable factors associated with an increased rate of cognitive decline is critical, especially as cognitive impairment continues to be a major issue affecting a large proportion of older adults.

Cognitive impairment has been found to affect 20% of older adults aged 65 years and over, and 45% of older adults aged 90 and older [4]. Impaired cognition may reduce one’s ability to carry out instrumental activities of daily living (IADL), cope with chronic disease symptoms, perform self-care and adhere to medication instructions, leading to a lower quality of life [5,6]. Moreover, cognitive impairment may exert a long-term impact on cognition and has been found to be associated with a more degenerative course of illness in terms of depression, cardiovascular events and mortality than was previously assumed [7,8,9,10]. Thus, with a rapidly growing older population in China, the high prevalence and burden of cognitive impairment emphasize the critical need to conduct research on the prevention of cognitive impairment among older adults.

Previous research has found that social relationship factors such as social isolation have been shown to be associated with cognitive impairment [11,12,13,14]. This previous work has generally indicated that social isolation has a negative effect on cognitive function and is even associated with increased risk of dementia. However, limited evidence focuses on the role of loneliness, a broadly useful indicator of perceived isolation, which has been defined as the perception of a deficit between the desired and actual quality and quantity of support received within the social context [15]. Recent evidence has indicated that loneliness, but not social isolation, was associated with poorer cognitive function or dementia [16,17]; yet another study identified no significant relationship between these factors [18]. Due to the mixed results yielded by different studies, the determination of whether loneliness is associated with cognitive impairment is vital and warrants further study to develop effective intervention programs and policies, aiming to protect older adults from cognitive impairment.

Beyond age, sex or gender is a major predictor of the development of Alzheimer’s disease and related dementia. For example, data from the Framingham study was used to estimate the ‘remaining lifetime risk’ of developing Alzheimer’s disease among those aged 65, finding a nearly two-fold risk among females (12%) relative to males (6.3%) [19]. Thus, identifying the role of sex in assessing the rate of cognitive decline is critical. Furthermore, sex warrants a closer examination in China, where women often have less access to education in early life, and less access to skills development and labor market opportunities than men in adult life, which might be due to sex-based discrimination and women’s roles of being a caretaker in a traditional social setting. Women’s socio-economic disadvantages were found to be harmful to cognitive development and maintenance [20], leading to a higher risk of cognitive impairment as compared to men in late-life [21,22]. In addition, although most studies found that women were more likely to feel lonely than men, a recent study reported intensity of lonely feelings was lower in women, which might be explained by the larger social network [23], and more emotional and intimate relationships [24]. Men who felt lonely had a greater risk of adverse mental health conditions than women [25]. Thus, investigating whether the association between loneliness and the risk of cognitive impairment varies by sex was warranted. This is a timely issue, given greater insight will inform specific and effective interventions to reduce the risk of cognitive impairment among older adults reporting feelings of loneliness. However, to our knowledge, insufficient evidence has explored sex differences between loneliness and cognitive impairment in China.

This study aims to help close this gap in the existing literature by evaluating the association between loneliness and cognitive impairment among older men and women in China using a nationally representative longitudinal sample. We hypothesized that loneliness would serve as a longitudinal predictor of cognitive impairment after the adjustment for other cognition-related factors.

## 2. Materials and Methods 

### 2.1. Data

The data used in the study were derived from the Chinese Longitudinal Healthy Longevity Survey (CLHLS), which is the largest longitudinal survey of the oldest-old ever conducted in a developing country. The CLHLS, a collaborative project between Duke University in the United States and Peking University in China, was conducted using a sample (randomly selected) pulled from 22 provinces in China. This survey was first administered in 1998 and originally targeted only the oldest-old (aged above 80 years). Subsequent follow-up surveys were carried out in 2000, 2002, 2005, 2008/2009 and 2011/2012. The CLHLS is an ongoing project, and a variety of mechanisms are in place to ensure the quality of the CLHLS data. Details regarding the sampling design and data quality mechanisms can be found elsewhere (Zeng, 2008). The survey was approved by Duke University Health System’s Institutional Review Board (IRB00001052-13074), and informed consent was obtained from each respondent.

The assessment criteria for cognitive impairment used in the latest two waves (2008/2009 and 2011/2012 waves) were different from those used previously, with some important confounders, such as cardiovascular disease (CVD) and diabetes, having a high rate of missing data before the 2008/2009 wave. Thus, the most recent waves (2008/2009 and 2011/2012; denoted as baseline and follow-up, respectively) of the CLHLS were used in this study. Longitudinal data were obtained for 16,954 respondents. At follow-up, 2894 (17.07%) participants were lost to follow-up and 5635 (33.24%) participants had died. Of the remaining 8425 participants, we excluded 132 (1.57%) who were younger than 65 years of age at baseline and 1395 (16.82%) who had cognitive impairments at baseline. Ultimately, 6898 qualified participants were included in the present study.

### 2.2. Measures

#### 2.2.1. Cognitive Impairment

Cognitive function was measured by a self or proxy-report at follow-up using a culturally adapted, Chinese version of the Mini-Mental State Examination (MMSE) that had been translated from the international standard of the MMSE questionnaire [26] and carefully tested via pilot survey interviews. The Chinese version of the MMSE consists of the following five dimensions: Orientation capacity, reaction, calculation ability, recall, and language ability [27]. As recommended by previous studies, responses indicating the participant was “unable to answer” were regarded as incorrect answers [28]. Based on the questionnaire responses, an aggregated score ranging from 0 to 30 is calculated, with higher scores indicating better cognitive ability. More detailed descriptions for the Chinese version of MMSE and its scoring system have been published elsewhere [28,29]. As previously described [28], to reduce MMSE measurement error, a score of less than 18 was used to categorize subjects as cognitively impaired, as approximately 90% of the respondents had received less than six years of schooling.

#### 2.2.2. Loneliness

At baseline, loneliness was defined using the following question: “Do you feel lonely?” This item was extracted from the Center for Epidemiologic Studies Depression Scale (CES-D) [30]. Potential responses included “never, rarely, sometimes, often, and always”. Many previous studies have used this indicator, the results of which suggest that this item may serve as a direct indicator of the feeling of loneliness [16,18,31]. For the purpose of statistical analysis, we dichotomized the feeling of loneliness variable into the following two groups: “Lonely” (sometimes, often and always) and “not lonely” (rarely and never) [32].

#### 2.2.3. Covariates

Data for all covariates, which were categorized into four sets, were acquired at baseline. The following social-demographic variables were included in the first set: Age, education level, employment status, and body mass index (BMI). Age was measured in chronological years and was grouped into 10-year increments. Educational attainment or education level was categorized as illiterate, primary school, middle school, high school and college or above. As employment status was likely to be associated with cognitive function [33], we controlled for current employment status as a dichotomous variable. Those with BMIs between 18.5 to 24.0 kg/m^2^ were categorized as being of normal weight, while others were defined as having an abnormal BMI. The second set included indicators of social isolation, which was assessed using the following three separate items: Living alone (yes or no), being married (yes or no) and having social support [16]. Social support was a four-question index based on the social ties perceived when sick, talking about daily life, expressing their thoughts, and seeking help [34]. Lack of social support was defined as having no support from others when the aforementioned four circumstances occurred.

The analyses were also adjusted for lifestyle variables, such as current smoking and current drinking, which were categorized as yes or no. Exercise was measured by the question “Do you exercise regularly at present?” (yes or no). The final set included health status, such as CVD, diabetes and activities of daily living (ADL) disability. CVD and diabetes were self-reported or proxy-reported and dichotomized as yes or no. ADL consisted of basic ADL (BADL) and instrumental ADL (IADL). BADL was measured based on the following six daily self-care tasks: Dressing, eating, toileting, bathing, indoor activities, and continence [35]. The IADL scale included the following eight daily tasks: Taking public transportation, shopping, visiting neighbors, washing clothing, cooking a meal, lifting a weight of 5 kg, walking continuously for 1 km and continuously crouching and standing up three times [36]. Disability in BADL or IADL was defined as the inability to independently perform any of the corresponding tasks. Consequently, in this study, if a respondent was disabled in BADL, IADL or both, he/she was defined as ADL disabled.

### 2.3. Statistical Analyses

Arithmetic means and standard deviations were used to describe continuous variables, while frequency distributions were calculated for categorical variables. A logistic regression analysis was performed to determine whether there were sex differences in the association observed between loneliness at baseline and cognitive impairment at follow-up. Three models were developed for both men and women in this study. Model 1 included socio-demographic factors (age, sex, education, marital status, engagement in physical labor, and type of residence), lifestyle factors (smoking, drinking and exercise) were added in model 2. Then, in model 3, the model was further adjusted for the health status (ADL disability, hypertension, diabetes, and CVD). For all performed regression analyses, we reported odds ratios (OR) with corresponding 95% confidence intervals (95% CI). Analyses were carried out using Stata version 13.0 (Stata Corp; College Station, TX, USA). Multiple imputations were applied to address missing data (for variables imputed: Median percentage missing 11.16%, minimum 0.04% and maximum 33.88%) using the NORM program [37,38].

## 3. Results

Table 1 presents the summary statistics of the variables. The numbers of men and women were comparable, with slightly more women included than men. Overall, 22.9% of the male and 30.6% of the female respondents reported suffering from loneliness. The rate of cognitive impairment observed among elderly women (22.6%) at follow-up was more than twice that identified among their male counterparts (11.0%). There was a female predominance in the oldest-old group, and the proportion of women aged 95 years and over was more than twice that of the proportion of men in this age group. Half of the men had received primary school education, while most of the women (73.8%) were illiterate. Approximately 20% of men were employed, a rate that was twice that observed among women. The average baseline cognitive function score of the male participants was 25.8, which was slightly higher than the baseline cognitive function score identified among female respondents. Notably, 67.5% of women were single at baseline. However, the proportion of men who reported being single was much lower. More women than men had an ADL disability. The rate of disability was 37.7% and 58.2% among men and women, respectively.

The results of the analysis of loneliness and cognitive impairment among older men are displayed in Table 2. Baseline loneliness demonstrated a stable and significant association with cognitive impairment at follow-up, as did age, education level, employment status, baseline cognitive function, marital status and ADL disability. A 30% increase in the odds of cognitive impairment was observed among those with loneliness (OR = 1.30; 95% CI 1.01–1.69). Our findings also showed that older age was significantly associated with cognitive impairment (OR = 1.85; 95% CI 1.59–2.16). Education served as a protective factor for cognitive function. Among older adults, the receipt of more education was associated with a lower odds of cognitive impairment (OR = 0.78; 95% CI 0.68–0.90). In addition, a significant association between employment status and cognitive impairment was observed. The odds of cognitive impairment were lower among participants who were employed (OR = 0.34; 95% CI 0.19–0.61). Baseline cognitive function was significantly associated with cognitive impairment. Higher cognitive function at baseline was associated with lower odds of cognitive impairment (OR = 0.93; 95% CI 0.89–0.97). Those who had a spouse were less likely to have impaired cognition (OR = 0.68; 95% CI 0.52–0.89). ADL disability was another factor that was closely associated with cognitive impairment. The odds of being cognitively impaired were almost twice as great among those with an ADL disability as those without (OR = 1.79; 95% CI 1.36–2.35).

Table 3 presents the results of the analysis of the association between loneliness and cognitive impairment among female older adults. A noteworthy finding was that baseline loneliness was not associated with follow-up cognitive impairment among participating women, regardless of the model employed; however, older age, lower education level, unemployment, lower baseline cognition, having no spouse and being ADL disabled were negatively associated with cognitive impairment at follow-up. Being older was associated with more than twice the odds of cognitive impairment (OR = 2.14; 95% CI 1.91–2.39). The odds of cognitive impairment were lower when respondents had a higher education level (OR = 0.62; 95% CI 0.51–0.76), were employed (OR = 0.57; 95% CI 0.34–0.94) and had a spouse (OR = 0.67; 95% CI 0.51–0.87). Furthermore, being ADL disabled was associated with increased odds of follow-up cognitive impairment among women (OR = 1.60; 95% CI 1.26–2.01).

## 4. Discussion

The study examined the association between loneliness and cognitive impairment among elderly men and women in China. The results showed that loneliness was significantly associated with cognitive impairment among the population of elderly men but not among the population of elderly women. This study expands upon the current body of literature, as this is the first study to our knowledge that assessed separate sex differences along the study timeline in the association between loneliness and cognitive impairment using data from a nationally representative longitudinal survey conducted in China.

Our results were consistent with existing research on the association between loneliness and cognitive decline or function. Bao-Liang Zhong (2016) reported that both transient and chronic loneliness were significant predictors of cognitive decline among members of the CLHLS cohort over the course of a six-year follow-up period [39]. A major distinction between this earlier work and the current study was that the current study separated analyses by sex and therefore allowed for exploration into factors associated with cognitive impairment separately, rather than only adjusting for the variable in a model combining males and females as in prior work. Thus, this study contributes uniquely to the existing literature about the distinct experience of males and females, respectively. The results of other research conducted by Bao-Liang Zhong (2017) further supported this relationship, indicating that loneliness may be associated with poor cognitive function [17]. Our findings underscored the detrimental role of loneliness on cognitive impairment, which may also be explained by recent findings [40]. First, analysis of neuroendocrine effects indicated that loneliness was associated with higher concentrations of cortisol, leading to dysregulation of the hypothalamic-pituitary adrenocortical axis, which contributes to inflammatory processes that play a role in hypertension, atherosclerosis and coronary heart diseases. Second, in terms of genetic effects, loneliness might have a unique transcriptional influence with potential health relevance. Third, studies of immune function have suggested that loneliness was associated with lower natural killer cell activity and poorer antibody response [41,42]. Ultimately, these data implied that the relationship between loneliness and cognitive functioning could be associated with too much or too little activity of certain neurotransmitters and hormones, leading to brain damage [43].

The current study adds to previous studies by showing that loneliness had a significant impact on cognitive impairment among elderly men, but not women. One of the possible explanations for the non-significant association observed between loneliness and cognitive impairment among elderly women was that women might have a greater capacity to insulate themselves against loneliness than men. Men may be encouraged to demonstrate taciturnity and predominantly seek social contact in the form of public organizations, the members of which might not always serve as confidants [44]. Women, however, may have more multifaceted social networks, including close friends and neighbors [25]. Cognitive reserve theory states that social networks affects brain structure, such as neurogenesis [45,46], and an increase of synaptic density, leading to more efficient use of brain networks [46]. Another explanation for this finding was that the experience of loneliness has been reported to have a stronger association with psychological distress, such as more depressed or lower satisfaction, among men than women [25,47]. The findings of a brain autopsy of a diseased sample in Wilson et al. (2003) suggested that psychological distress was associated with higher risk of cognitive impairment, including Alzheimer’s disease, through neurobiological mechanisms [48]. Finally, previous research also indicated that the feeling of loneliness may lead to stress, which could bring about an inflammatory response in men only [49]. As a result, increased inflammatory reaction may deteriorate mental health, including cognitive functioning [50]. These findings indicate that public policies that target loneliness and its associated factors may effectively decrease cognitive impairment among elderly men and, in turn, promote good health among the elderly and lessen the burden of cognitive impairment that can extend, in many ways, to their caregivers, friends, family, and, by extension, society as a whole.

Some limitations of the current study are worth noting. First, the use of a validated scale to identify loneliness might have improved the accuracy of its measurement relative to the use of a single question. However, the single loneliness question has been reported to be more appropriate for use in the oldest age group than the UCLA loneliness scale [31,51]. Second, as the CLHLS originally targeted the oldest-old in China, approximately 50% of the respondents at baseline were lost to follow-up or died before the 2011/2012 wave, which may bias the results. However, the participants who were deceased or loss to follow-up were more likely to be less educated, older, having more chronic conditions or disabled in ADL at baseline, which were harmful for cognitive health. Therefore, the estimated association between loneliness and cognitive impairment may be prone to a downward bias in the present study [52]. Third, as cognitive impairment has a long-lasting preclinical stage [53], the potential for reverse causality cannot be completely removed due to the relatively short follow-up period. However, a prospective, longitudinal design was used in the present study by excluding individuals who had cognitive impairment at baseline while also controlling for multiple chronic diseases in the analysis, which leads to lessening the potential bias resulting from reverse causality [54]. Finally, mental health variables, such as depression are not included in the CLHLS. As such, it was not possible to test the sex differences in mental health by adding the interaction items between loneliness and mental health in the current analysis. Future studies are needed to further explain our understanding of the sex differences in the relationship between loneliness and cognitive impairment.

## 5. Conclusions

As a developing country and a rapidly aging society, China has increasing populations of both older adults and individuals that are cognitively impaired. Focusing on cognitive impairment and its modifiable risk factors may contribute to achieving the strategic targets for healthy aging. The current study revealed that loneliness was a risk factor for cognitive impairment among elderly men. Although elderly women more frequently reported feelings of loneliness, the impact of loneliness on cognitive impairment was significant among elderly men but not elderly women. These findings should be considered when developing strategies to protect against loneliness in later life. Given interventions to reduce loneliness have been found to be effective in the prevention of cognitive impairment, further work in this area is likely to have important and substantial public health benefits in terms of cognitive impairment and dementia screening, prevention, and control, particularly among elderly men.

## Figures and Tables

**Table 1:** Baseline characteristics of the study sample (*N* = 6898; %).

Variables		Men (*N* = 3390)	Women (*N* = 3508)
Loneliness	Lonely	22.9	30.6
	Not lonely	77.1	69.4
Age (year)	~65	35.2	30.3
	~75	32.0	29.2
	~85	26.6	27.6
	~95	6.2	12.9
Education level	Illiterate	29.3	73.8
	Primary school	50.0	20.5
	Middle school	11.0	3.4
	High school	6.3	1.6
	College or above	3.4	0.7
Working status	Still working	19.6	9.5
	Not working	80.4	90.5
BMI	Normal	61.3	53.2
	Abnormal	38.7	46.8
Baseline cognitive functioning ^a^	–	25.8 (2.6)	24.7 (2.9)
Marital status	Having a spouse	64.4	32.5
	No spouse	35.6	67.5
Living alone	Yes	13.9	19.5
	No	86.1	80.5
Social support	Yes	92.6	93.3
	No	7.4	6.7
Smoking	Yes	38.1	6.2
	No	61.9	93.8
Drinking	Yes	34.0	7.8
	No	66.0	92.2
Exercises	Yes	42.2	30.4
	No	57.8	69.6
Diabetes	Yes	3.0	3.0
	No	97.0	97.0
CVD	Yes	27.3	31.5
	No	72.7	68.5
ADL disability	Yes	37.7	58.2
	No	62.3	41.8

^a^ Mean (standard deviation) was used to present the continuous variable. BMI = body mass index; CVD = cardiovascular diseases and ADL = activities of daily living.

**Table 2:** Association between loneliness and cognitive impairment among men aged 65 or older.

Independent Variables	Cognitive Impairment					
	Model 1		Model 2		Model 3	
	OR	95% CI	OR	95% CI	OR	95% CI
Loneliness	1.39 *	1. 08–1.80	1.37 *	1. 50–1.80	1.30 *	1.01–1.69
Age	2.09 ***	1.81–2.42	2.04 ***	1.76–2.36	1.85 ***	1.59–2.16
Education level	0.76 ***	0.66–0.88	0.77 ***	0.67–0.89	0.78 **	0.68–0.90
Working status	0.30 ***	0.17–0.52	0.30 ***	0.17–0.53	0.34 ***	0.19–0.61
BMI	1.14	0.90–1.45	1.14	0.91–1.46	1.17	0.92–1.48
Baseline cognitive functioning	0.91 ***	0.88–0.95	0.92 ***	0.88–0.95	0.93 ***	0.89–0.97
Married	0.67 **	0.51–0.87	0.67 **	0.51–0.87	0.68 **	0.52–0.89
Living alone	0.81	0.58–1.14	0.81	0.58–1. 13	0.85	0.60–1.19
Social support	0.83	0.54–1.28	0.85	0.55–1.31	0.88	0.57–1.35
Smoking			0.81	0.62–1.05	0.81	0.62–1.06
Drinking			0.81	0.62–1.05	0.83	0.63–1.08
Exercises			0.76 *	0.60–0.98	0.81	0.63–1.03
Diabetes					0.55	0.21–1.39
CVD					1.03	0.79–1.34
ADL disability					1.79 ***	1.36–2.35

* *p* < 0.05; ** *p* < 0.01 and *** *p* < 0.001. BMI = body mass index; CVD = cardiovascular diseases and ADL = activities of daily living.

**Table 3:** Association between loneliness and cognitive impairment among women aged 65 or older.

Independent Variables	Cognitive Impairment					
	Model 1		Model 2		Model 3	
	OR	95% CI	OR	95% CI	OR	95% CI
Loneliness	1.01	0.83–1.22	0.99	0.82–1.21	0.98	0.81–1.19
Age	2.32 ***	2.09–2.58	2.32 ***	2.08–2.58	2.14 ***	1.91–2.39
Education level	0.61 ***	0.50–0.74	0.62 ***	0.51–0.75	0.62 ***	0.51–0.76
Working status	0.52 ***	0.32–0.86	0.52 *	0.31–0.86	0.57 ***	0.34–0.94
BMI	1.06	0.89–1.26	1.06	0.89–1.27	1.07	0.89–1.27
Baseline cognitive functioning	0.94 ***	0.91–0.97	0.94 ***	0.91–0.97	0.95 **	0.92–0.98
Married	0.66 **	0.50–0.85	0.65 **	0.50–0.84	0.67 **	0.51–0.87
Living alone	0.80	0.64–1.01	0.81	0.64–1.02	0.83	0.66–1.05
Social support	0.95	0.67–1.34	0.96	0.68–1.35	0.95	0.67–1.35
Smoking			0.86	0.58–1.28	0.85	0.57–1.26
Drinking			0.72	0.51–1.02	0.72	0.51–1.02
Exercises			0.85	0.69–1.04	0.88	0.72–1.09
Diabetes					0.61	0.28–1.32
CVD					0.87	0.71–1.06
ADL disability					1.60 ***	1.26–2.01

* *p* < 0.05; ** *p* < 0.01 and *** *p* < 0.001. BMI = body mass index; CVD = cardiovascular diseases and ADL = activities of daily living.
